# Epidemiology, treatment patterns, clinical outcomes, and disease burden among patients with immune‐mediated thrombotic thrombocytopenic purpura in the United States

**DOI:** 10.1002/rth2.12802

**Published:** 2022-09-16

**Authors:** Ayoade Adeyemi, Francesca Razakariasa, Alexandra Chiorean, Rui de Passos Sousa

**Affiliations:** ^1^ Sanofi Cambridge Massachusetts USA; ^2^ Quinten France Paris France; ^3^ Sanofi Lisbon Portugal; ^4^ Present address: Alexion Boston Massachusetts USA; ^5^ Present address: Krystal Biotech Zug Switzerland

**Keywords:** blood platelets, epidemiology, morbidity, thrombotic microangiopathies, thrombotic thrombocytopenic purpura

## Abstract

**Background:**

Immune‐mediated thrombotic thrombocytopenic purpura (iTTP) is a life‐threatening thrombotic microangiopathy. Due to its rarity, epidemiology and real‐world outcomes data are scarce.

**Objectives:**

The aim was to assess epidemiology, treatment patterns, clinical outcomes, and disease burden in patients with iTTP in the United States.

**Methods:**

This longitudinal, retrospective observational study of the Optum‐Humedica database included patients with an iTTP diagnosis (≥1 documented ADAMTS13 activity less than 10% or one or more iTTP episodes) from January 2007 to December 2019.

**Results:**

Of 666 patients with an iTTP diagnosis between October 2015 and December 2019, 302 (45%) had one or more iTTP episodes. The pooled annual incidence of documented iTTP during this period was 3.43/million, and the annual incidence of one or more iTTP episodes was 1.81/million. Patients with one or more iTTP episodes received a median of six therapeutic plasma exchange (TPE) sessions per episode; 86% received corticosteroids, and 59% received rituximab. Exacerbations occurred in 17% (52/302) and relapse in 11% (34/302); 34% (103/302) had one or more thromboembolic events. Mortality rates during the study period were 25% (167/666) among all patients with iTTP diagnosis, and 14% (41/302) among patients with one or more iTTP episodes. In the assessment of disease burden (January 2007 to September 2019), patients in the iTTP cohort (*n* = 514) presented with a mean of 14 comorbidities, compared with 3 in a matched non‐iTTP cohort (*n* = 2570). In a cluster analysis, duration of iTTP episode and mortality rate were greater in older versus younger patients.

**Conclusions:**

Despite treatment with TPE and immunosuppressants, patients with iTTP have high risk of morbidity and mortality, demonstrating the need for more effective therapies.

**Essentials**

- Real‐world data on outcomes in immune‐mediated TTP (iTTP) are limited.
- Database study among patients in the United States with iTTP prior to wider availability of caplacizumab.
- Despite treatment, patients with iTTP have a high risk of morbidity and death.
- More effective therapies are needed to improve clinical outcomes.

## 1 INTRODUCTION

Immune‐mediated thrombotic thrombocytopenic purpura (iTTP), also known as acquired thrombotic thrombocytopenic purpura, is a rare, life‐threatening thrombotic microangiopathy (TMA) with a global annual incidence of 1–2 cases per 1 million people.^1^ iTTP is characterized by hemolytic anemia, severe thrombocytopenia, and organ damage. The clinical presentation is heterogeneous^2^ and reflects ischemic damage to organs, mainly the heart, brain, and gastrointestinal tract, and the kidneys in some cases.^1^, ^2^, ^3^, ^4^ Without prompt treatment, multiorgan failure and death can occur within days of an acute iTTP episode.^2^, ^5^

Historically, management of iTTP has been based on therapeutic plasma exchange (TPE) and immunosuppressive therapy (corticosteroids and, increasingly, rituximab).^1^ However, even with this treatment regimen, episodes of iTTP are associated with acute mortality rates of 8%–20% and a relapse rate of greater than 30%.^4^, ^5^, ^6^ Approximately 30%–50% of patients experience exacerbations,^7^ nearly 30% have TPE‐related complications,^8^ and 10%–20% are refractory to treatment.^9^, ^10^, ^11^, ^12^, ^13^ In addition, over long‐term follow‐up, patients in recovery from iTTP have increased morbidity and deficits in health‐related quality of life and are at greater risk for cognitive difficulties, depression, hypertension, stroke, and autoimmune disease.^14^, ^15^, ^16^ Persistent organ damage may be caused by ischemia upon microvascular thrombosis during acute episodes of iTTP.^14^ Therefore, there is a need to rapidly inhibit microthrombosis to minimize organ damage.

Caplacizumab is a von Willebrand factor (VWF)‐directed Nanobody® that rapidly inhibits the interaction of VWF with platelets and reduces the pathophysiological formation of microthrombi.^17^ Caplacizumab in conjunction with TPE and immunosuppression was approved for the treatment of iTTP by the US Food and Drug Administration in February 2019 and became commercially available in the United States in April 2019.^18^ The International Society on Thrombosis and Haemostasis guidelines recommend using caplacizumab over not using caplacizumab for patients with a high pretest probability of iTTP and access to a disintegrin and metalloproteinase with a thrombospondin type 1 motif, member 13 (ADAMTS13) activity test results within 72 h.^19^

Owing to the rarity and heterogeneity of the disease, data on iTTP epidemiology, disease management, and clinical outcomes are often varied and scarce, including in the United States. We therefore conducted a study using a large electronic health record (EHR) database to assess real‐world epidemiology, treatment patterns, and clinical outcomes, and associated disease burden among patients with iTTP in the United States, prior to the wider availability of caplacizumab as a treatment option.

## 2 METHODS

### 2.1 Study design and database

**FIGURE 1:**
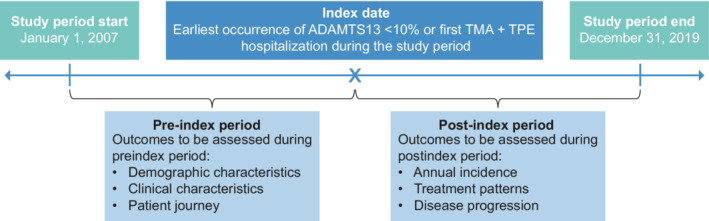
Study design. Study periods were October 2015–December 2019 (Objectives 1 and 2) and January 2007–September 2019 (Objective 3). ADAMTS13, a disintegrin and metalloproteinase with a thrombospondin type 1 motif, member 13; TMA, thrombotic microangiopathy; TPE, therapeutic plasma exchange.

This was a longitudinal, retrospective, observational cohort study based on de‐identified data from patients with an iTTP diagnosis within the longitudinal EHR repository from Optum, Optum‐Humedica (Figure 1). This repository includes more than 700 hospitals and 7000 clinics in the United States, treating more than 95 million patients. Clinical, claims, and other administrative data are obtained from both inpatient and ambulatory EHRs, practice management systems, and other internal systems, and are processed, normalized, and standardized across both acute inpatient stays and outpatient visits. Data elements include demographics, medications prescribed and administered, immunizations, allergies, lab results, vital signs, clinical and inpatient stay administrative data, and coded diagnoses and procedures. For mortality data, in addition to those obtained from inpatient claims, data are also sourced from the Social Security Administration–Death Master File and obituary data from Datavant. The Optum‐Humedica database was accessed through licensing agreements with Optum.

The study objectives were to (i) describe the annual incidence of iTTP and the annual incidence rate of iTTP episodes; (ii) describe treatment patterns and clinical outcomes among patients with an iTTP diagnosis; and (iii) characterize the disease burden outside episode‐related hospitalizations by identifying patterns of iTTP‐related comorbidities.

### 2.2 Study population

For the annual incidence, treatment patterns and clinical outcomes (Objectives 1 and 2), patients were selected if, between October 2015 and December 2019, they had either one or more documented ADAMTS13 test result less than 10% or one or more documented iTTP episodes, defined as one or more inpatient stays with an International Classification of Diseases (ICD) diagnosis of TMA (ICD, Ninth Revision [ICD‐9], dx: 446.6; ICD, Tenth Revision [ICD‐10], dx: M31.1), and one or more TPE procedures (Current Procedural Terminology‐4 code: 36514; ICD‐10: 6A551Z3) during the same inpatient stay. Patients were excluded if they had a medical encounter related to conditions that mimic iTTP on or 6 months before the index date (systemic infection [including severe sepsis], Rocky Mountain spotted fever, aspergillosis infection, hypertensive crisis, or *Escherichia coli* infection during the index hospitalization); human immunodeficiency virus (HIV), organ/stem cell transplant, and malignancy, anytime 6 months prior to or during the index hospitalization; or hemolytic‐uremic syndrome during the study period. This approach of selecting patients with iTTP has been used in previous database studies.^20^, ^21^ The study period of October 2015 to December 2019 was selected to use the latest data available, and to include only ICD‐10 codes to more accurately identify the iTTP patient population, and to improve the estimation of incidence (ICD‐10 codes were released in October 2015).

For the disease burden outside episode‐related hospitalization (Objective 3), a longer study period (from January 2007 to September 2019) was used to adequately capture substantive data on disease burden. Patients without 12 months or more of preindex and 12 months or more of postindex data were excluded. A control cohort of patients without a diagnosis of iTTP in the Optum database was matched with the iTTP group by age, gender, and index year at a ratio of 1:5.

### 2.3 Outcomes and analyses

The index date was defined as the date of the earliest occurrence of an ADAMTS13 test result less than 10% or first TMA + TPE‐related hospitalization during the study period. Index iTTP episode was defined as the first occurrence of TMA + TPE‐related hospitalization. Patients were followed from the index date until loss to follow‐up, end of study period, or death, whichever occurred first.

The following outcomes were analyzed descriptively (Objectives 1 and 2): demographic and clinical characteristics of patients; annual incidence of iTTP; incidence rate of iTTP episodes; treatments received; and treatment‐related clinical outcomes (exacerbation, relapse, thromboembolic [TE] events, mortality). Descriptive statistics included mean, standard deviation (SD), and median values for continuous variables, and frequency for categorical variables. Incidence was calculated from the number of patients with a newly documented iTTP diagnosis during a specific year divided by the total number of patients during that year, multiplied by 1 million. Pooled annual incidences were estimated using data from 4 full years (2016–2019). Exacerbations were defined as rehospitalization for TPE within the first 30 days following discharge for the index episode. Relapse was defined as a new iTTP episode more than 30 days after discharge for the index episode.

To characterize the disease burden (Objective 3), 29 potential iTTP‐related symptoms and conditions, which we refer to in this analysis as comorbidities, were identified from the literature and/or based on insights from medical experts.^22^, ^23^, ^24^ Diagnosis, procedure codes, labs, signs, and disease and symptom terms and treatments were used to identify the select comorbidities from the database. The prevalence of these comorbidities was compared between patients with iTTP and a matched control cohort using Cramer's V association score analysis. All comorbidities occurring during an iTTP episode‐related hospitalization were removed from the analysis to focus on the long‐term disease progression. Thereafter, homogenous subgroups of patients with iTTP were identified, such that patients were clustered using a K‐mode approach based on demographic features (sex, age, race) and clinical features (number of episodes, acute and episodic symptoms outside an episode, iTTP‐related comorbidities outside an episode). The optimal number of clusters (*n* = 4) with small subgroup variances was obtained based on cost function. To assess the consistency of the characteristics of the four clusters identified in the base‐case results, a sensitivity analysis was conducted using data from October 2015 to December 2019, the time period used for Objectives 1 and 2. Finally, PrefixSpan, a sequential pattern mining method,^25^ was used to identify patterns in iTTP‐related comorbidities in the overall iTTP cohort and by cluster; this was to better understand the entire iTTP population and improve the significance of the iTTP‐related comorbidities identified.

Methods to minimize bias were not applicable in this descriptive study. Missing data were not imputed and were reported as is for all analyses.

## 3 RESULTS

### 3.1 Annual incidence of iTTP and iTTP episodes, treatment patterns and clinical outcomes (Objectives 1 and 2)

#### 3.1.1 Analysis population and patient characteristics

**FIGURE 2:**
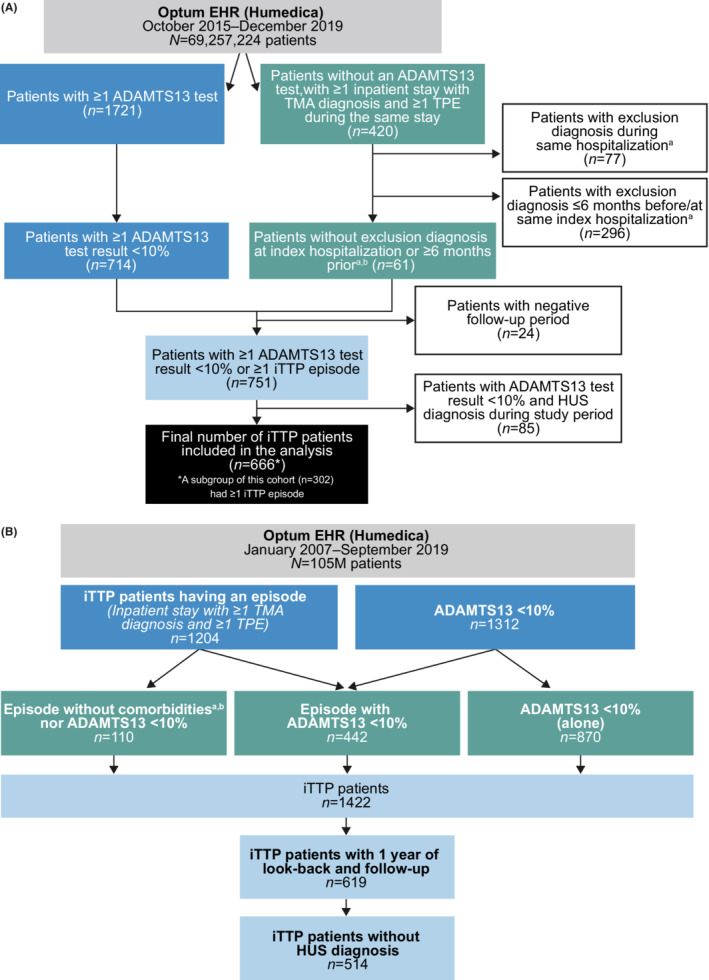
Patient flow through the study for Objectives 1 and 2 (A) and Objective 3 (B). ADAMTS13, a disintegrin and metalloproteinase with a thrombospondin type 1 motif, member 13; EHR, electronic health record; HIV, human immunodeficiency virus; HUS, hemolytic uremic syndrome; iTTP, immune‐mediated thrombotic thrombocytopenic purpura; TMA, thrombotic microangiopathy; TPE, therapeutic plasma exchange. ^a^HUS, systemic infection, Rocky Mountain spotted fever, aspergillosis infection, *Escherichia coli* infection, or hypertensive crisis during same hospitalization. ^b^HIV, organ/stem cell transplant, malignancy, or systemic lupus erythematosus ≤6 months before or during same index hospitalization.

For Objectives 1 and 2, a total of 666 patients met inclusion criteria for iTTP diagnosis (Figure 2A). In this cohort of patients with iTTP diagnosis, median (interquartile range [IQR]) age was 50 (35–63) years, and 63% were women (Table 1). Median (IQR) follow‐up duration was 10.8 (1.9–26.2) months. More than half of patients (58%) had an Elixhauser Comorbidity Index score of 3 or greater (Table 1).

**TABLE 1:** Baseline demographic and clinical characteristics of patients with iTTP

	All patients with iTTP diagnosis (*n* = 666)	Patients with ≥1 iTTP episode (*n* = 302)
Age at index date, years		
Mean (SD)	49.2 (18.7)	46.2 (16.2)
Median (IQR)	50 (35–63)	45 (33–57)
Females, *n* (%)	421 (63)	219 (73)
Race, *n* (%)		
White	380 (57)	150 (50)
African American	214 (32)	116 (38)
Asian	10 (2)	5 (2)
Other or unknown	62 (9)	31 (10)
BMI,^a^ kg/m^2^		
Mean (SD)	29.1 (7.0)	31.2 (6.7)
Median (IQR)	28.8 (24.1–33.1)	30.5 (27.2–35.1)
Elixhauser Comorbidity Index categories,^b^ *n* (%)		
0	115 (23)	89 (39)
1	49 (10)	27 (12)
2	43 (9)	26 (11)
≥3	288 (58)	86 (38)
Follow‐up duration, months		
Median (IQR)	10.8 (1.9–26.2)	ND
Range	0–51.6	ND
Patients with ≥12 months of follow‐up, *n* (%)	316 (47%)	ND

Abbreviations: BMI, body mass index; IQR, interquartile range; iTTP, immune‐mediate thrombotic thrombocytopenic purpura; ND, not determined; SD, standard deviation.

^a^ Prior to, but closest to, the index date; *n* = 435 for all patients with iTTP diagnosis, *n* = 146 for patients with ≥1 iTTP episode.

^b^ Among patients with ≥12 months of baseline period.

Among the 666 patients with iTTP diagnosis, 302 patients had one or more iTTP episodes. In this group of patients, median (IQR) age was 45 (33–57) years, 73% were women, and 38% had an Elixhauser Comorbidity Index score of 3 or greater. The remaining 364 patients in the cohort of 666 patients with iTTP diagnosis did not have a documented iTTP episode during the study period, but had ADAMTS13 test result <10%. These patients were likely in remission from an iTTP episode that occurred prior to the beginning of the study period.

#### 3.1.2 Incidence of iTTP and iTTP episodes

A mean of 145 patients/year were newly documented to present with iTTP diagnosis (ADAMTS13 less than 10% or an iTTP episode [TMA + TPE‐related hospitalization]) from January 2016 to December 2019 (203 patients per year in 2016, 151 in 2017, 134 in 2018, and 92 in 2019). The pooled annual incidence of documented iTTP (ADAMTS13 less than 10% or an iTTP episode) during this period was 3.43/million.

A total of 318 episodes of iTTP (56–90/year) were recorded between 2016 and 2019. Based on the subgroup of patients with one or more iTTP episodes, the pooled annual incidence of one or more iTTP episodes (averaged over this period) was 1.81/million.

#### 3.1.3 Treatment patterns and clinical outcomes

Treatment patterns: Therapies received by all patients with an iTTP diagnosis and in patients with one or more iTTP episode are summarized in Table 2. Patients with one or more iTTP episodes received a median (IQR) of 6 (3–13) TPE sessions per episode; the proportions of patients using corticosteroids, rituximab, other immunosuppressant medications, and caplacizumab were 86%, 59%, 7%, and 1%, respectively. Non–iTTP‐related treatments received by all patients with an iTTP diagnosis and among patients with one or more iTTP episodes included anticoagulants (10% and 12%), antidepressants (13% and 14%), anxiolytics (52% and 61%), respectively, and antihypertensives (4% each).

**TABLE 2:** Treatment patterns among patients with iTTP during the follow‐up period

	All patients with iTTP diagnosis (*n* = 666)	Patients with ≥1 iTTP episode (*n* = 302)
iTTP‐related treatments		
TPE, *n* (%)	329 (49)	302 (100)
Mean (SD) number of TPE sessions per patient	15.9 (21.7)	16.7 (22.3)
Median (IQR) number of TPE sessions per patient	9 (3–20)	10 (4–21)
Mean (SD) number of TPE sessions per episode	NA	11.0 (15.6)^a^
Median (IQR) number of TPE sessions per episode	NA	6 (3–13)
Corticosteroids, *n* (%)	406 (61)	261 (86)
Rituximab, *n* (%)	210 (32)	178 (59)
Other immunosuppressants, *n* (%)	51 (8)	22 (7)
Caplacizumab,^b^ *n* (%)	4 (1)	4 (1)
Non–iTTP‐related treatments, *n* (%)		
Anticoagulants	67 (10)	35 (12)
Antidepressants	87 (13)	41 (14)
Anxiolytics	343 (52)	185 (61)
Antihypertensives	24 (4)	13 (4)

Abbreviations: IQR, interquartile range; iTTP, immune‐mediated thrombotic thrombocytopenic purpura; NA, not applicable; SD, standard deviation; TPE, therapeutic plasma exchange.

^a^ Based on a total of 351 episodes among the 302 patients.

^b^ Available for clinical use in the United States since April 2019.

Clinical outcomes: Among patients with one or more iTTP episodes, exacerbations (rehospitalizations for TPE within the first 30 days following discharge for any iTTP episode) occurred in 17% (52/302). Relapse (new iTTP episode more than 30 days after discharge for the index episode) was reported in 11% (34/302).

At least one TE event was reported in 21% of patients with an iTTP diagnosis and 34% of patients with one or more iTTP episodes. Cerebral infarction and myocardial infarction were the most frequently reported TE events in both groups (Table 3).

**TABLE 3:** Clinical outcomes among patients with iTTP during the post‐index period

	All patients with iTTP diagnosis (*n* = 666)	Patients with ≥1 iTTP episode (*n* = 302)
Exacerbation, *n* (%)	N/A	52 (17)
Relapse, *n* (%)	N/A	34 (11)
Patients with iTTP episode, *n* (%)^a^		
≥1	302 (45)	302 (100)
≥2	34 (5)	34 (11)
≥3	9 (1)	9 (3)
≥4	3 (<1)	3 (1)
≥5	3 (<1)	3 (1)
Patients with ≥1 TE event,^b^ *n* (%)	139 (21)	103 (34)
Total number of TE events	193	152
TE type, *n* (%; of total number of TE events)	–	–
Cerebral infarction	106 (55)	77 (51)
Myocardial infarction	37 (19)	32 (21)
Vascular syndromes	20 (10)	17 (11)
Pulmonary embolism	15 (8)	12 (8)
Arterial embolism	7 (4)	7 (5)
Portal vein obstruction	5 (3)	4 (3)
Deep vein thrombosis	2 (1)	2 (1)
Mesenteric vein embolism	1 (1)	1 (1)
Deaths, *n* (%)	167 (25)	41 (14)
Mean (SD) time to death from index date, days	176.6 (289.1)	246.6 (360.0)
Median (IQR) time to death from index date, days	33 (13–178)	90 (23–579)
Death within 45 days of index iTTP episode, *n* (% of total deaths)	21 (13)	21 (51)
Deaths based on time from index date (months)		
0–6	129 (77)	28 (68)
7–12	11 (7)	3 (7)
13–18	7 (4)	3 (7)
19–24	6 (4)	2 (5)
25–30	7 (4)	NA
31–36	5 (3)	NA
37–42	1 (1)	NA
43–48	1 (1)	NA
Mean (SD) age at death, years	60.6 (15.4)	59.3 (15.6)
Median (IQR) age at death, years	64 (52–71)	61 (48–70)

Abbreviations: IQR, interquartile range; iTTP, immune‐mediated thrombotic thrombocytopenic purpura; NA, not available; SD, standard deviation; TE, thromboembolic.

^a^ Includes the index episode and any iTTP episode occurring during the follow‐up period.

^b^ Occurring during the postindex period.

Mortality rate during the follow‐up period among all patients with an iTTP diagnosis was 25%, with most deaths (77%) occurring within 6 months of the index date. Among patients with one or more iTTP episodes, 14% died during the follow‐up period. Around half of these deaths (51%) occurred within 45 days of the index episode; 68% occurred within 6 months of the index date (Table 3).

### 3.2 Patterns of iTTP‐related comorbidities (Objective 3)

#### 3.2.1 Analysis population and patient characteristics

For Objective 3, a total of 514 patients met inclusion criteria for iTTP diagnosis and had 12 or more months of pre‐ and postindex follow‐up data (Figure 2B). In this cohort of patients with iTTP, median (IQR) age was 49 (36–62) years, and 70% were women. These patients were matched to a control cohort of 2570 patients without iTTP. Median (IQR) duration of coverage (period between the start of the preindex period and the end of postindex follow‐up) was 9 (7–12) years.

#### 3.2.2 Comorbidities in the iTTP versus control cohort

Among 29 prespecified comorbidities, patients with iTTP presented with a mean of 14 comorbidities during the observed period (outside of episode‐related hospitalizations), compared with only 3 comorbidities per patient in the control cohort (Table 4).

**TABLE 4:** Comorbidities in the iTTP cohort versus control cohort

Comorbidity	iTTP (*n* = 514)		Control (*n* = 2570)		Chi‐square	Cramer
	*n* (%)	Mean age of occurrence (years)	*n* (%)	Mean age of occurrence (years)		
ECG abnormalities	157 (30.5)	52	174 (6.8)	60	<0.0001	0.28
Adult jaundice	65 (12.6)	45	17 (0.7)	48	<0.0001	0.27
Altered mental status	198 (38.5)	54	130 (5.1)	65	<0.0001	0.40
Anxiety	277 (53.9)	47	555 (21.6)	47	<0.0001	0.27
Aphasia	59 (11.5)	57	24 (0.9)	72	<0.0001	0.24
Arrhythmia	309 (60.1)	51	374 (14.6)	60	<0.0001	0.41
Chest pain	229 (44.6)	49	457 (17.8)	53	<0.0001	0.24
Chronic fatigue	140 (27.2)	50	223 (8.7)	50	<0.0001	0.21
Chronic kidney disease	243 (47.3)	54	181 (7.0)	64	<0.0001	0.43
Coronary syndrome and myocardial infarction	126 (24.5)	56	117 (4.6)	65	<0.0001	0.27
Deep vein thrombosis	45 (8.8)	55	13 (0.5)	65	<0.0001	0.22
Depression	232 (45.1)	48	498 (19.4)	48	<0.0001	0.22
Dizziness/giddiness	284 (55.3)	50	391 (15.2)	52	<0.0001	0.36
Dysarthria	48 (9.3)	58	15 (0.6)	71	<0.0001	0.23
Dyspnea	297 (57.8)	50	422 (16.4)	56	<0.0001	0.36
End‐stage renal disease	93 (18.1)	50	13 (0.5)	63	<0.0001	0.36
Epilepsy/seizures	94 (18.3)	48	73 (2.8)	48	<0.0001	0.25
Facial weakness	17 (3.3)	54	16 (0.6)	68	<0.0001	0.09
Gross hematuria	94 (18.3)	49	121 (4.7)	54	<0.0001	0.20
Headache	315 (61.3)	46	588 (22.9)	46	<0.0001	0.31
Hypertension	359 (69.8)	51	817 (31.8)	58	<0.0001	0.29
Personality change	6 (1.2)	60	11 (0.4)	53	0.0818	0.03
Proteinuria	123 (23.9)	48	37 (1.4)	52	<0.0001	0.38
Pulmonary embolism	86 (16.7)	53	42 (1.6)	60	<0.0001	0.28
Speech language	99 (19.3)	53	42 (1.6)	65	<0.0001	0.31
Stroke	200 (38.9)	53	164 (6.4)	62	<0.0001	0.37
TIA	80 (15.6)	55	76 (3.0)	66	<0.0001	0.21
Tingling	209 (40.7)	47	306 (11.9)	52	<0.0001	0.29
Walking difficulties	100 (19.5)	55	126 (4.9)	65	<0.0001	0.21

Abbreviations: ECG, electrocardiogram; iTTP, immune‐mediated thrombotic thrombocytopenic purpura; TIA, transient ischemic attack.

Cramer's V score for the 29 iTTP‐related comorbidities was consistently higher for patients with iTTP compared with the control cohort, indicating a stronger correlation. The comorbidities with the greatest differences in prevalence between the iTTP and control cohorts included renal diagnosis of chronic kidney disease (CKD; 47% vs. 7%), proteinuria (24% vs. 1.4%), and end‐stage renal disease (18% vs. 0.5%); neurological signs and symptoms with altered mental status (39% vs. 5%), stroke (39% vs. 6%), dizziness/giddiness (55% vs. 15%), speech and language difficulties (19% vs. 2%), and tingling (41% vs. 12%); cardiovascular diseases (CVDs) with arrhythmia (60% vs. 15%) and hypertension (70% vs. 32%); and respiratory symptoms such as dyspnea (58% vs. 16%); all of these differences were statistically significant (*p* < 0.05).

#### 3.2.3 Sequence of symptoms/comorbidities

**FIGURE 3:**
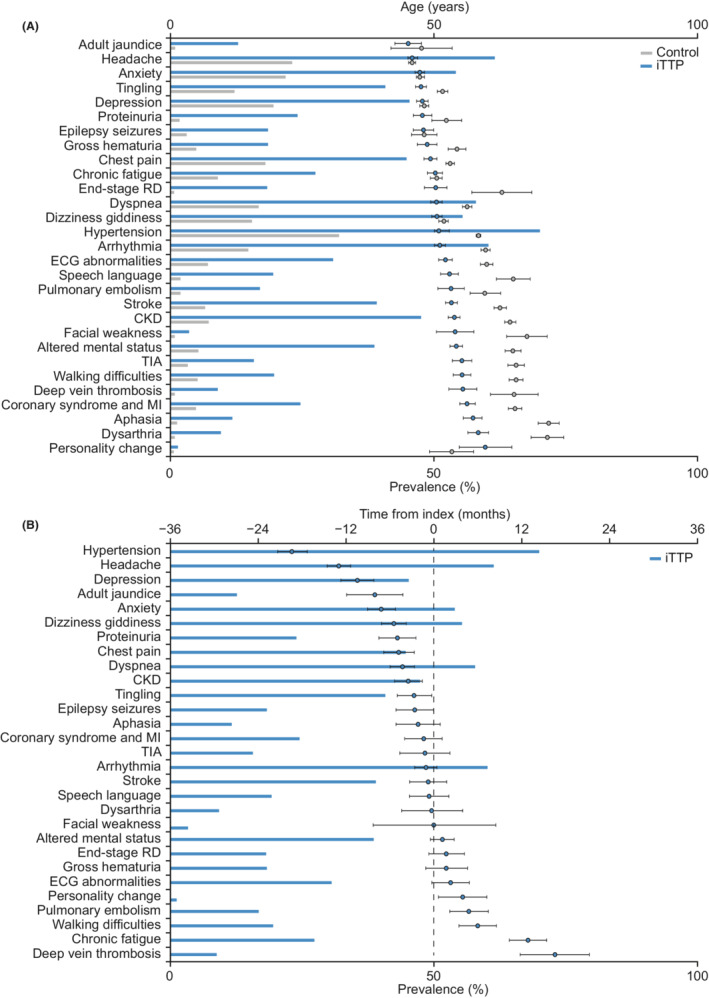
Comparison of comorbidities in the iTTP versus control cohort: (A) Prevalence of comorbidities and age of occurrence (ranked from earliest to latest) and (B) time difference between comorbidity occurrence and index date^a^. ^a^Negative values indicate onset prior to index date; positive values indicate onset after index date. CKD, chronic kidney disease; ECG, electrocardiogram; iTTP, acquired thrombotic thrombocytopenic purpura; MI, myocardial infarction; RD, renal disease; TIA, transient ischemic attack

In the overall iTTP cohort, comorbidities were observed in the following order based on their average age of appearance: general symptoms (headache, anxiety), renal disease, CVD, and neurological symptoms (particularly associated with stroke and transient ischemic attack) (Figure 3A). Renal diseases, CVD, and neurological symptoms occurred at an earlier age in the iTTP cohort compared with the average age of occurrence of these comorbidities in the control cohort. In contrast, headache, anxiety, epilepsy, and depression occurred at about the same age in both cohorts.

In the iTTP cohort, 10 of 29 comorbidities had a mean time of onset after the index date of iTTP diagnosis (Figure 3B). The earliest comorbidities, hypertension and headache, presented more than 12 months before the index date. The next comorbidities (occurring within 12 months before the index date) included mood changes, dizziness, chest pain and breathlessness, and renal problems (eg, proteinuria, CKD). Around the time of the index date, patients presented with cardiovascular symptoms and events (coronary syndrome and myocardial infarction, transient ischemic attack, arrhythmia, stroke) followed by speech and language difficulties, facial weakness, altered mental status, and further renal dysfunction. Following the index date, patients experienced comorbidities such as fatigue, walking difficulties, and TE events.

Pattern mining in the overall iTTP cohort identified 64 sequences comprising four distinct comorbidities, with each sequence found in 52–76 patients. The most common conditions and comorbidities in the sequences were mild, including hypertension, headache, chest pain, dyspnea, dizziness/giddiness, and anxiety. A total of 10 sequences ended with stroke.

#### 3.2.4 Cluster analysis

**FIGURE 4:**
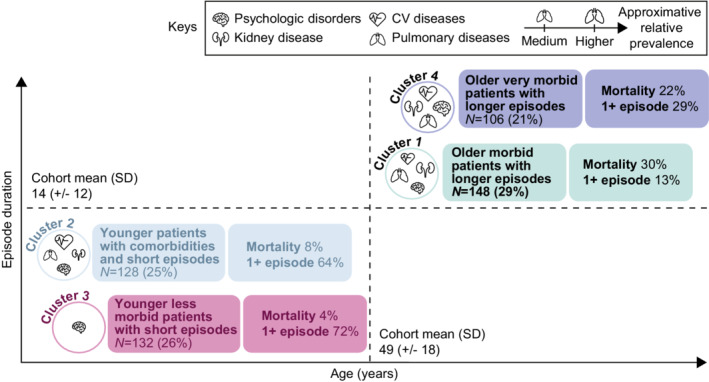
Baseline demographic and clinical characteristics of patients with iTTP by cluster. CV, cardiovascular; iTTP, acquired thrombotic thrombocytopenic purpura; SD, standard deviation.

Cluster analysis of the iTTP cohort based on observed patterns in demographic and clinical characteristics (including comorbidities) identified four clusters, each representing 21%–29% of the iTTP cohort (Figure 4, Figure S1).

##### Cluster 1: “Older morbid patients with longer episodes”

This cluster consisted of patients with a mean age of 56 years; 60% were women. This cluster had the highest mortality (30%) and a mean duration of index iTTP episode of 19 days, despite a low rate of having one or more iTTP episodes (13%). There was a high burden of renal diseases, with a high prevalence of proteinuria (34%), CKD (66%), and end‐stage renal disease (23%). In addition, 60%, 71%, and 85% of patients had dyspnea, arrhythmia, and hypertension, respectively. Although patients from this cluster were relatively less burdened than patients of similar age from Cluster 4 for neurological conditions, signs of altered mental status and dizziness/giddiness were reported in 40% and 39% of patients, respectively.

##### Cluster 2: “Younger patients with comorbidities and short episodes”

Patients in this cluster were younger (mean age, 40 years), and more than 80% of patients were women. This cluster had a low mortality rate (8%) and a shorter mean duration of index iTTP episode of 13 days compared with Cluster 1, but with a high rate of having one or more reported iTTP episodes (64%). This cluster was largely burdened by CVD, with hypertension, arrhythmia, and chest pain reported by 70%, 65%, and 65%, respectively. Other common comorbidities were headache (86%), chronic fatigue (37%), anxiety (76%), and depression (67%). Neurological conditions were less frequent, except for dizziness/giddiness (80%) and tingling (68%).

##### Cluster 3: “Younger less morbid patients with short episodes”

This cluster of younger patients (mean age, 43 years; 76% were women) had the lowest mortality rate (4%) and a short mean duration of the index iTTP episode of 12 days. Cluster 3 had the highest proportion of patients with one or more iTTP episodes (72%). The mean number of iTTP‐related comorbidities per patient was seven, the lowest among the clusters; nearly one‐third suffered from stroke.

##### Cluster 4: “Older very morbid patients with longer episodes”

Patients in this cluster were the oldest (mean age, 58 years), with a smaller proportion of female patients (62%) compared with the overall cohort (70%). Despite having a lower rate of ≥1 episode (29%), this cluster had a high mortality rate (22% vs. 16% in overall cohort) and the longest mean index episode duration (20 days) of all four clusters. This cluster of patients had a mean of 22 comorbidities, the highest among all clusters, and was most impacted by CVD (71% of patients had coronary syndrome and myocardial infarction), respiratory disorders (91% had dyspnea), and renal diseases (86% reported CKD). Neurological manifestations were also frequent in this cluster, with 69% experiencing one or more stroke events (vs. 39% for overall cohort). Transient ischemic attack occurred in 33% of patients; potential consequences included walking difficulties (43%), speech and language impairment (38%), and altered mental status (79%).

Results of a sensitivity analysis involving a shorter study period (October 2015 to December 2019) were consistent with the overall base‐case cluster analysis.

There was a distinct sequence of appearance of comorbidities in each cluster (Figures S2 and S3). In Cluster 1 (older, morbid patients), renal dysfunction occurred first, followed by cardiovascular and neurological symptoms. Patients from this cluster appeared to encounter severe comorbidities earlier in the order of occurrence compared with patients from Cluster 4. In Cluster 4 (older, very morbid patients), mild symptoms appeared first, followed by more severe symptoms (stroke, CKD, embolism). In Cluster 2 (younger, mostly female morbid patients), symptoms and comorbidities with a high prevalence (such as headache, anxiety, chest pain, dyspnea, and arrhythmia) appeared at a relatively young age. In Cluster 3 (younger, mostly female less morbid patients), end‐stage renal disease appeared first and approximately 10 years earlier than in other iTTP patients, followed by symptoms with high prevalence rates (eg, depression, anxiety, headache).

## 4 DISCUSSION

This study evaluated the incidence, treatment patterns, clinical outcomes, and disease burden associated with iTTP within the US patient population prior to the wider availability of caplacizumab as a treatment option, and as such, only 1% of the overall population was treated with this innovative therapy. Based on a large, real‐world database, the incidence of iTTP episodes in the United States was 1.81/million. Despite treatment of these acute episodes with TPE and immunosuppression, rates of exacerbation, relapse, mortality, and TE events were high, demonstrating the need for early diagnosis, prompt treatment, and more effective therapies to improve clinical outcomes in patients with iTTP. Furthermore, patients with iTTP had a greater number of comorbidities compared with a control cohort without iTTP, reflecting a high disease burden.

The outcomes of patients with iTTP in our study were largely consistent with previous findings. Real‐world data from 2010 suggest an estimated 40% of patients are at risk of relapse within 7.5 years of an initial iTTP episode.^6^ While the relapse rate observed in this study was lower, that is, 11% over a shorter follow‐up period of 4 years, there is an ongoing unmet need in relapse prevention among patients experiencing one or more iTTP episodes. The observed mortality rate of 14% among patients with one or more iTTP episodes is consistent with the 8%–20% reported in the literature for patients treated with TPE and immunosuppression.^2^, ^6^, ^26^, ^27^ More recently, survival rates of over 95% have been reported for patients receiving the latest recommended standard of care of TPE, immunosuppression, and caplacizumab.^9^, ^28^ Caplacizumab became available in the United States in April 2019, which was toward the end of the study period. This may explain why only four patients in our cohort of patients with iTTP diagnosis received caplacizumab during the captured time period (October 2015 to December 2019). Therefore, these findings mainly reflect historical therapies and the associated unmet medical need. Future studies are warranted to evaluate the impact of caplacizumab on mortality rates in US real‐world practice.

Occurrence of complications such as microvascular ischemia during acute iTTP episodes may translate to persistent long‐term organ damage. A growing body of evidence in the literature demonstrates that the consequences of iTTP last beyond the acute episode. For example, in an analysis of the Oklahoma TTP‐HUS Registry, patients who had recovered from an acute iTTP episode were at higher risk for hypertension, systemic lupus erythematosus, and premature death compared with US and Oklahoma reference populations.^14^ In addition, in a survey from the United Kingdom, patients who had experienced iTTP reported impairments in health‐related quality of life, cognitive function, psychological well‐being, and physical functioning, highlighting the need for long‐term care after iTTP.^29^ Consistent with the potential of iTTP to have lasting effects on patients beyond the acute episode, in our study, iTTP was associated with significant comorbidities, many of which appear to have been precipitated by iTTP, such as renal disease, cardiovascular disease, and neurological symptoms. It should be noted that a higher prevalence of renal disease was observed in this study in comparison with prior studies.^30^ Further studies are needed to evaluate these potential long‐lasting impacts of iTTP on patient health.

Cluster analysis identified patients who are at risk for poor outcomes and may help guide disease management based on specific clinical characteristics or patterns of comorbidities. Our study revealed a higher mortality rate in older versus younger patients with iTTP, suggesting that iTTP may pose a greater and more severe burden of disease on older patients, who may already present with other unrelated preexisting comorbid conditions. According to a registry study in Italy, elderly patients with iTTP (65 years of age or older), compared with age‐matched individuals without iTTP, had a higher prevalence of multimorbidity and polypharmacy.^31^ Additionally, consistent with our cluster analysis, a registry study from France reported that older patients with iTTP (60 years of age or older) presented with more comorbidities (particularly CVD and cancer), were more likely to receive antihypertensive treatment, antiplatelet therapy, and vitamin K antagonists, and had higher short‐ and long‐term mortality rates compared with younger patients (less than 60 years of age).^32^ Together, these findings underscore the need for further caution in the management of elderly patients with iTTP.

Overall, this study strengthens the evidence that treatment gaps exist for iTTP and highlights the suboptimal outcomes in patients with iTTP. Our findings suggest that iTTP is associated with greater prevalence of comorbidities. However, the following study limitations should be noted. EHR data are primarily collected for medical purposes rather than research and therefore are prone to incomplete or inaccurate coding of diagnoses. This could lead to misclassification or underreporting of baseline characteristics, comorbidities, or outcomes. For patients identified by ADAMTS13 test, the database may not have captured the initial or any prior iTTP episodes, leading to the inclusion of patients without reported episodes in the cohort. This may have contributed to the higher median age observed (50 years) in patients with iTTP diagnosis identified in this study, as nearly half of these patients were identified by ADAMTS13 test and were likely to be in remission from an iTTP episode that occurred prior to the beginning of the study. Also, for patients identified by a documented iTTP episode, the first reported episode in the database may not have been the first in the patient's life. Data on treatment are based on prescriptions issued; it is unknown whether all prescriptions were recorded accurately or if the patient used the medication. Further, the small proportion of patients who received caplacizumab may be a limitation of the Optum database, as higher proportions were observed in other databases using a similar time period (unpublished observation). Another important limitation was that mortality data were obtained from Social Security Administration–Death Master File, providers' notes, and obituary notes; studies using this method have been found to underestimate mortality. As data on cause of death were not available, it is possible that not all deaths captured were iTTP related. It is important to note that this study was not designed to report clinical outcomes related to any treatment; iTTP outcomes in this study were based on records of a TMA diagnosis and TPE during inpatient stays, rather than on their lactate dehydrogenase or platelet count profiles. In addition, as the Optum database is limited to commercially insured patients, study findings may not be generalizable to the wider iTTP population; additional studies may be needed to validate these findings across other data sources and patient populations. Nevertheless, the longitudinal EHR repository includes data from more than 7000 health care settings across the United States, representing more than 95 million patients.

In summary, this real‐world analysis of a large US health records database found high mortality and morbidity in patients with iTTP, despite treatment with TPE and immunosuppression, demonstrating the need for more effective therapies to improve clinical outcomes. Comorbidities precipitated by iTTP may reflect long‐term organ damage, highlighting the need for rapid control of ischemia during the acute episode. Furthermore, clinical outcomes and disease burden worsen with increasing age, as evidenced by the onset of more severe symptoms and higher mortality within the clusters with older patients. A better understanding of the sequence and pattern of comorbidities may translate to improved recognition, monitoring, and management of comorbidities in patients with iTTP.

## AUTHOR CONTRIBUTIONS

AA contributed to data analysis/interpretation and critical revision of the manuscript for important intellectual content. RDPS contributed to study conception and design, data acquisition, data analysis/interpretation, and critical revision of the manuscript for important intellectual content. FR and AC contributed to data analysis/interpretation and critical revision of the manuscript for important intellectual content. All authors had access to the data, had full editorial control of the manuscript, and provided their final approval of all content.

## RELATIONSHIP DISCLOSURE

AA and RDPS were employees of Sanofi at the time research was conducted and may hold shares and/or stock options in the company. FR and AC have no potential conflicts of interest to declare.

## FUNDING INFORMATION

The study was funded by Sanofi.

## ETHICS APPROVAL AND PATIENT CONSENT

The Optum Humedica electronic health records database is fully compliant with the Health Insurance Portability and Accountability Act of 1996 (HIPAA). All data were de‐identified prior to acquisition, negating the need for institutional review board approval.

## Supporting information


**Figure S1:** Overview of baseline demographic and clinical characteristics of patients with iTTP by cluster

**Figure S2:** Related comorbidities, age of appearance and prevalence for (A) cluster 1 (older morbid patients with longer episodes) and (B) cluster 4 (older very morbid patients with longer episodes)

**Figure S3:** Related comorbidities, age of appearance and prevalence for (A) cluster 2 (younger patients with comorbidities and short episodes) and (B) cluster 3 (younger, less morbid patients with short episodes)

## Data Availability

Qualified researchers may request access to patient‐level data and related study documents, which may include the clinical study report, study protocol with any amendments, statistical analysis plan, and data set specifications. Patient‐level data are anonymized. Further details on Sanofi's data‐sharing criteria, eligible studies, and process for requesting access can be found at https://www.vivli.org/.
